# Summer and spring elbow rashes is a variant of polymorphous light eruption: confirmation by photoprovocation and histopathology in a series of five cases

**DOI:** 10.3389/fmed.2023.1260514

**Published:** 2023-10-19

**Authors:** María Victoria de Gálvez, Rosa Maria Castillo-Muñoz, Norberto López-Navarro, Patricio López-Jiménez, Enrique Navarrete-de Gálvez, Ricardo Bosch-García, Enrique Herrera-Acosta, José Aguilera

**Affiliations:** ^1^Photobiological Dermatology Laboratory, Medical Research Center, Department of Dermatology and Medicine, Faculty of Medicine, University of Málaga, Málaga, Spain; ^2^Dermatology Service, Hospital Clínico Universitario Virgen de la Victoria, Málaga, Spain; ^3^Dermatology Service, Hospital de Poniente, Almería, Spain; ^4^Project Engineering Area, Department of Graphic Expression Design and Projects, School of Engineering, University of Málaga, Málaga, Spain

**Keywords:** polymorphous light eruption, spring and summer eruption, ultraviolet A, phototest, photoprovocation, elbow

## Abstract

**Background:**

Summer and spring eruptions on the elbows are a variant of polymorphous light eruption described on clinical and histopathological grounds; however, to our knowledge, they have not been confirmed by photobiological studies.

**Objective:**

Based on photobiological studies, this study aimed to demonstrate the involvement of ultraviolet-A (UVA) radiation in this variant of polymorphous light eruption occurring exclusively on the elbows.

**Methods:**

A series of five patients with polymorphous light eruption lesions on the elbows were included in our study. All patients underwent phototesting and photoprovocation of the skin lesions after exposure to a UVA light source [Philips UVA HPA lamp (400 W)]. All patients underwent punch biopsy and histopathological and immunohistochemical studies with anti-CD123.

**Results:**

In all the cases, UVA irradiation caused the appearance of skin lesions on the elbows with characteristic polymorphous light eruption. Histological data showed edema in the superficial dermis and a perivascular lymphocytic infiltrate compatible with polymorphous light eruption. Immunohistochemical staining for CD1-23 showed negative results.

**Conclusions:**

For the first time, photobiological photoprovocation studies demonstrated that repeated exposure to UVA radiation leads to the generation of skin lesions on the elbows, which are clinically and histologically consistent with summer and spring eruptions, confirming that elbow rash is a variant of polymorphous light eruption.

## Introduction

Polymorphous light eruption (PLE) is a common idiopathic photodermatosis of unknown etiology. It has morphological variants, such as juvenile spring eruption, which mainly affects boys in the form of vesicles located on the pinnae (1). Polymorphous light eruption, which can manifest as a pinhead papular eruption on the face, has been described in patients with phototypes V-VI (2), as well as in forms of PLE without skin eruptions (3). Molina-Ruiz et al. (4) reported a relapsing eruption of the elbows, with histopathological and immunohistochemical findings of PLE. They considered it a localized variant of PLE and termed it “spring and summer eruption of the elbows.” A similar case was also reported by Curto-Barredo et al. in 2017 (5).

The etiopathogenesis of polymorphous light eruption has not been fully elucidated, although it is widely accepted that it is caused by a delayed hypersensitivity mechanism in response to UV-induced antigens that have not yet been identified (6, 7). A recent genomic-wide expression analysis showed that patients with PLE have a reduced expression of genes involved in cellular apoptosis processes, which could generate autoantigens (8). New advances in knowledge suggest that its pathogenesis might also involve immune system overactivation, which would escape UV-induced functional tolerance, as well as inflammatory alterations involving the microbiome (9). The latter has also been reported in recent studies that suggested that exposure to UV radiation may alter the microbiome and trigger PLE lesions (10, 11). Contrarily, cases of PLE have been observed in relatives of patients with lupus erythematosus, suggesting a shared pathogenesis (12).

PLE can be associated with other photodermatoses, such as solar urticaria. In both, visible light and UVA radiation (alone or in combination) are the most frequent triggers, although in some cases, a UVB-positive response has also been described (13). However, in exceptional cases, photoprovocation of lesions with UVA-promoted PLE and UVB radiation triggered SU in the same person (14, 15). The action spectrum of PLE is mostly attributed to UVA radiation, although ultraviolet-B radiation (UVB) and visible light may also be involved in some cases (15).

To our knowledge, the presence of elbow lesions as the sole manifestation of PLE has not yet been confirmed by photobiological tests. In our study, we observed five patients with spring and summer eruptions on the elbows and performed photobiological studies with UVA light emission sources to confirm their involvement in this localized PLE variant.

## Materials and methods

### Patients

**Figure 1:**
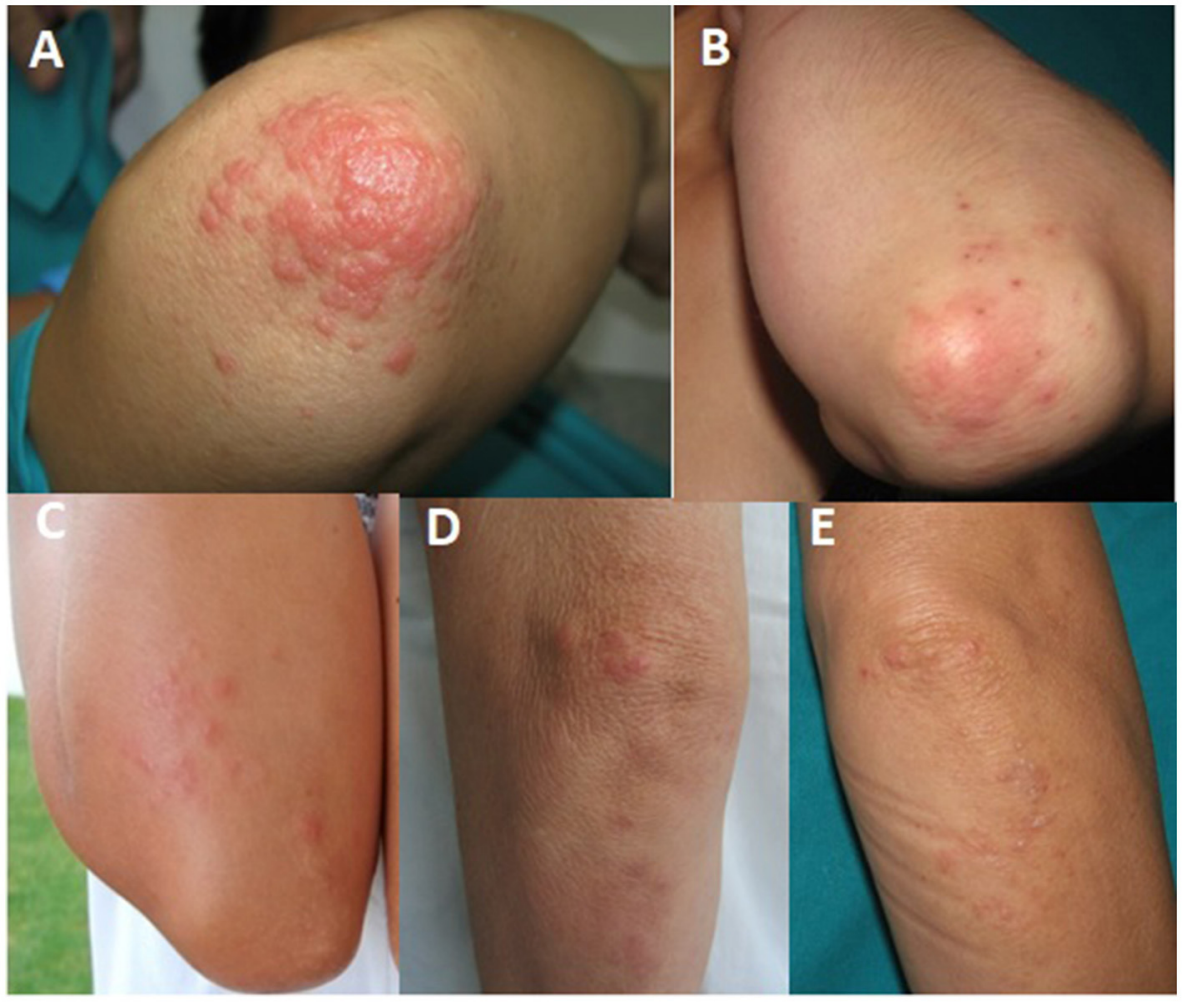
Clinical images of spring rash on the elbows in five patients who underwent photobiological studies. Some of the patients' photos **(B, C, E)** were self-taken before their visit to the hospital. They were asymptomatic during their first visit prior to undergoing photobiological studies. Patients **(A, D)** presented with some lesions during the first interview, as shown in the images.

An observational prospective study was performed on a total of five patients, four women and one man, diagnosed with possible polymorphous light eruption on the elbows. The patients were referred to the Dermatological Photobiology Laboratory of the Medical Research Center of the University of Malaga for photobiological studies. They were referred from two hospitals in Malaga, Spain (Hospital Clínico Universitario Virgen de la Victoria and Hospital Costa del Sol). The skin phototypes (Fitzpatrick) of the patients were as follows: one patient with phototype II, three patients with phototype III, and one patient with phototype IV. All patients reported a symmetrical, maculopapular, and pruritic skin eruption on the elbows that began in spring within hours/days of sun exposure and disappeared after several days without sun exposure, and topical treatment with corticosteroids was administered in some cases (Table 1; Figure 1). Subsequently, they presented similar recurrent outbreaks that were always related to sun exposure. All the patients studied had previously undergone blood tests, including porphyrins, IgE, and antinuclear antibodies, and the results were normal.

**Table 1:** Clinical data and lesions characteristics of patients included in the study.

**Case number**	**Sex/age**	**Phototype**	**Clinical evidences**	**Sistemic symtoms**	**Antinuclear antibody**	**Erythemal phototest (MED-mJ cm^−2^)**	**Abnormal reaction to UVA phototest**	**Photoprovocation UVA Dose (mJ cm^−2^)**	**Treatment**	**Evolution/start**
**1**	F/23	III	Erythematous papulovesicular vesicles on elbows; isolated lesions EESS and EEII	No	Negative	MED: 31 mJ cm^**–**2^	Negative	49.5 J cm^−2^	Spontaneous resolution within 3 weeks	3 years/spring
**2**	F/67	II	Erythematous papules and plaques on the elbows, isolated lesions on the back	No	Negative	MED: 31 mJ cm^**–**2^	Negative	43 J cm^−2^	Topycal corticoids	3 years/spring
**3**	F/56	IV	Erythematous papules on elbows	No	Negative	MED: 35.5 mJ cm^**–**2^	Negative	57 J cm^−2^	Spontaneous resolution within 2 weeks	2 years/spring
**4**	F/31	III	Erythematous macules, papules and plaques on elbo	No	Negative	MED: 31 mJ cm^**–**2^	Negative	49.5 J cm^−2^	Topycal corticoids	2 years/summer
**5**	M/21	III	Erythematous papules on elbows	No	Negative	MED: 23.2 mJ cm^**–**2^	Negative	43 J cm^−2^	Topycal cortids and antihistamins	3 years/summer

### Photobiological studies

All patients underwent a photobiological study consisting of phototesting and photoprovocation. No lesions were observed in any patient at the beginning of the photobiological study. The patients presented self-taken images of their lesions, and some of them presented lesion images taken during their visit to their attending dermatologist.

### Phototest

**Figure 2:**
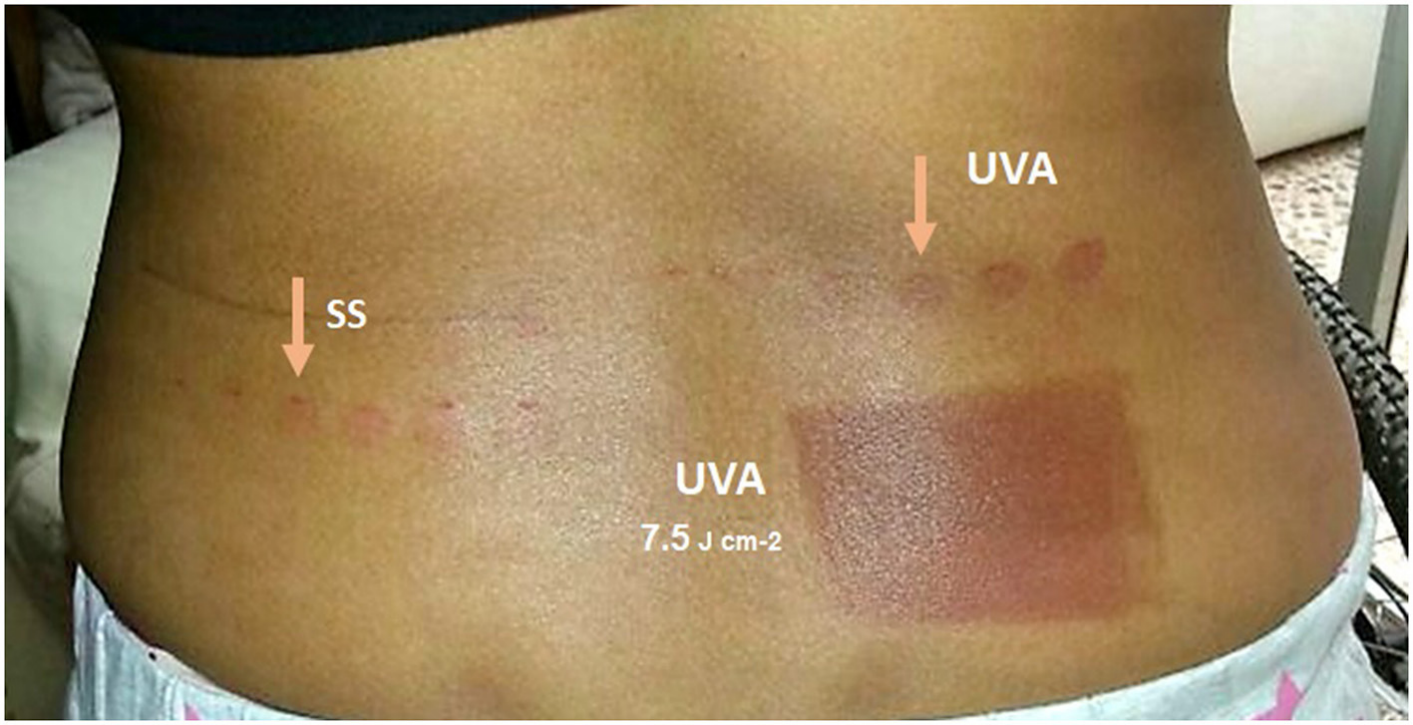
Image showing an example of the photobiological study. The image shows the back of Patient 3, taken 24 h after the photobiological study. On the left side, the erythema phototest was performed under the solar simulator (SS) with a minimum erythematous dose at the fourth point of the series (35.5 mJ cm^−2^). On the right side, an anomalous UVA phototest was performed with a minimal erythema-generating dose at the fourth point of the series (6 J cm^−2^). A similar reaction was observed on the lower back, with an area of intense erythema observed under UVA 24 h after the first UVA photoprovocation dose of 7.5 J cm^−2^.

First, a phototest was performed to analyze the skin's sensitivity to simulated solar UV radiation. For this purpose, the minimum erythema dose (MED) was first determined, which involved the exposure of six adjacent areas of 1.14 cm^2^ of the patients' backs to a series of increasing doses under a solar simulator (Oriel 300 W solar UV simulator, Newport Co. Nebraska, USA). The erythematous dose values were 9.2–12.3–15.5–23.2–31–35.5 mJ cm^−2^. Concurrently, similar to the MED phototest, an increasing dose series of UVA exposure was performed on each patient's skin to observe abnormal skin reactions to UVA (erythema instead of normal melanin pigmentation). For this, five adjacent areas of 1.14 cm^2^ each were irradiated with increasing doses (1.5–3–4.5–6–7.5–9 J cm^−2^) from under a high-pressure UVA light source (Philips UVA HPA-400 W, Philips, Eindhoven, the Netherlands). A double monochromator spectroradiometer fitted with an Ulbrich-type integrating sphere (Macam SR 9910 V7, Irradian Co, Scotland, UK) was used to measure the spectral irradiance of the solar simulator. The erythema reading was taken 24 h after irradiation. MED and abnormal UVA results were also read 24 h after irradiation (see Figure 2 for an example of the phototest and photoprovocation tests).

### Photoprovocation test

After the erythemal and UVA phototests, all patients were exposed to irradiation on two well-defined areas of at least 30 cm^2^ (the elbows and the back), with five repeated doses of 6–7.5–9–12–15 J cm^−2^ for five consecutive days (Monday–Friday); readings were taken daily and after the seventh day (Monday next week). To differentiate the exact localization of photoprovoked lesions, elbow irradiation always covered an area of 7–10 cm above and below the elbow joint. Some of the patients showed skin redness after 3 days of increased UV dosage. In these cases, the previous dose was repeated, and no increase in energy was made in the following days. All participants were asked to take photographs of the photoprovocation areas during the weekend.

### Histopathological and immunohistochemical studies

Photoprovoked lesions on the elbows of each patient were biopsied using a 4-mm punch biopsy tool for subsequent histopathological analysis. The samples were embedded in paraffin, and histological sections were stained with hematoxylin & eosin. The epidermal thickness, hyperkeratosis, dermo-epidermal junction (DEJ) changes, interstitial mucin deposition and pattern, and the inflammatory infiltrate type and density were evaluated.

In addition, a specific staining was performed to determine the anti-CD123 marker (Abcam Co. Cambridge, UK). Clusters were defined as nodular aggregates containing at least 10 PDCs. Scattered cells were defined as single PDCs distributed throughout the inflammatory infiltrate.

This study was approved by the Malaga Province Ethics Committee (EC:16-12-21-Consejería de Salud, Servicio Andaluz de Salud, Junta de Andalucía, Spain). All participating patients provided written informed consent for the photobiological studies.

## Results

### Photobiological studies

#### Phototest

The minimum erythematous dose observed in all patients ranged from 23.2 mJ cm^−^2 in patients with phototype II to 35.5 mJ cm^−^2 in the patient with phototype IV, indicating that the MEDs of all patients were within the normal range. Similarly, no abnormal reactions to UVA were observed 24 h after exposure to the different UVA doses in the series. Increased melanin pigmentation was observed at all points corresponding to a UVA dose of 7.5 J cm^−2^ in patients with phototypes II and III and to a UVA dose of 3 J cm^−2^ in the patient with phototype IV. No erythema was found under UVA in any of the five patients.

#### Fotoprovocation test

**Figure 3:**
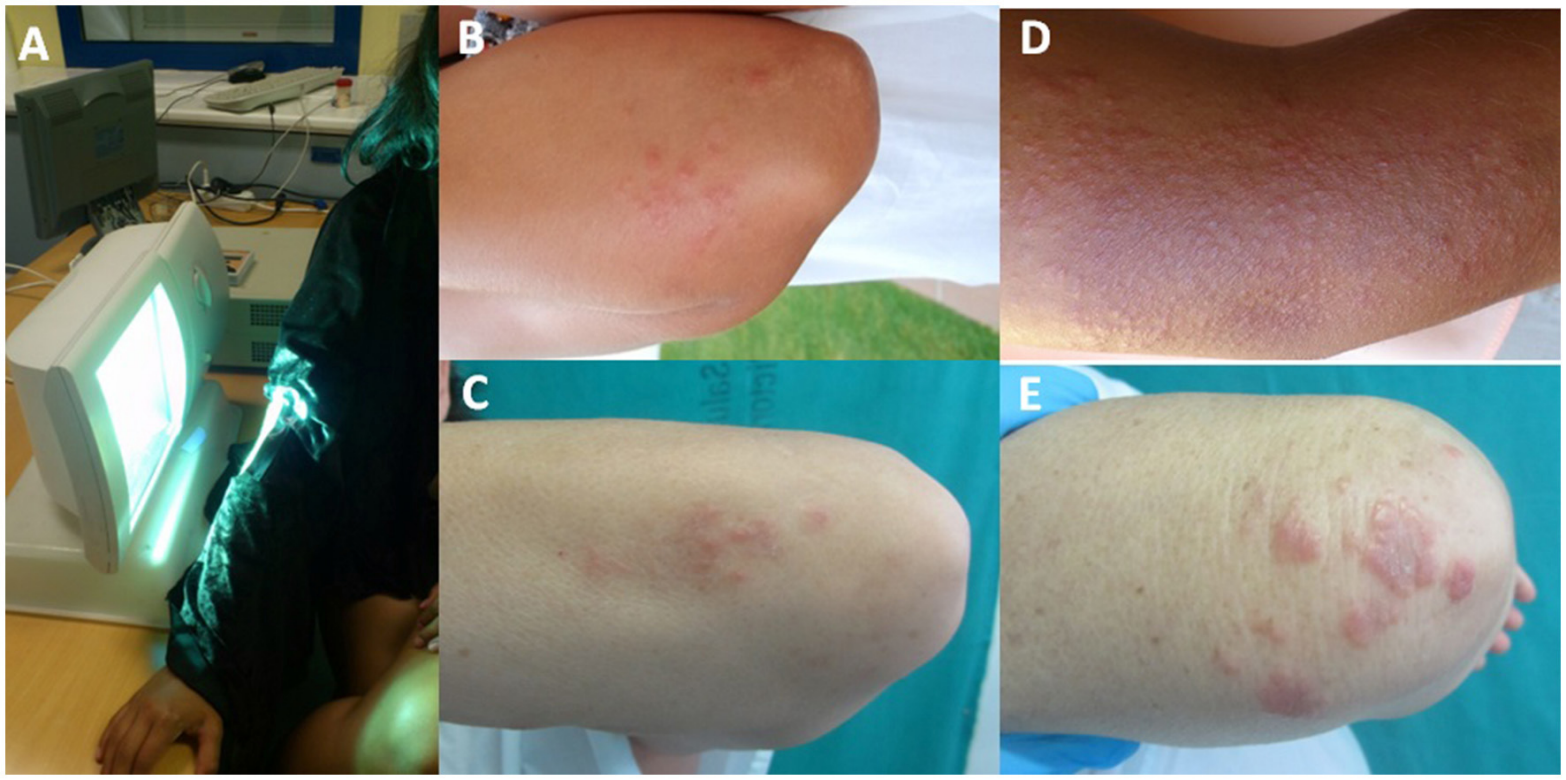
**(A)** Image of the UVA exposure of the elbow for photoprovocation tests on patients using a high-pressure UVA lamp. **(B, C)** Examples of the elbows at the beginning of the photoprovocation test for Patients 3 and 5. **(D)** Appearance of papular lesions grouped in plaques on the elbow against a hyperpigmented background after a photoprovocation series with a total dose of 57 J cm^−2^ in the case of Patient 3. **(E)** Image of papular lesions on Patient 5 after a total exposure of 43 J cm^−2^.

In all patients, the photoprovocation tests were positive on the elbows but not on the back. After each day of exposure, high melanin pigmentation was observed, and some level of redness due to UVA erythema was also observed in two of the patients after an accumulated dose of 15 J cm^−2^. The first skin photoprovoked lesions were observed on the fourth day in three of the patients and on the fifth day in the remaining two patients (31.5 and 49.5 J cm^−2^ of total UVA dose, respectively); the lesions were more evident in all the patients after the weekend (seventh day), with the appearance of papulo-erythematous lesions forming a plaque and accompanied by a lot of itching that lasted at least 1 week after the appearance, as can be seen in the images (Figure 3). Three of the five patients showed photoprovoked lesions 5 cm down the elbow joint in the forearm, while the remaining two patients showed lesions in the entire photoexposed area, as can be observed in Figure 3D.

#### Haematoxylin-eosin histopathological staining

**Figure 4:**
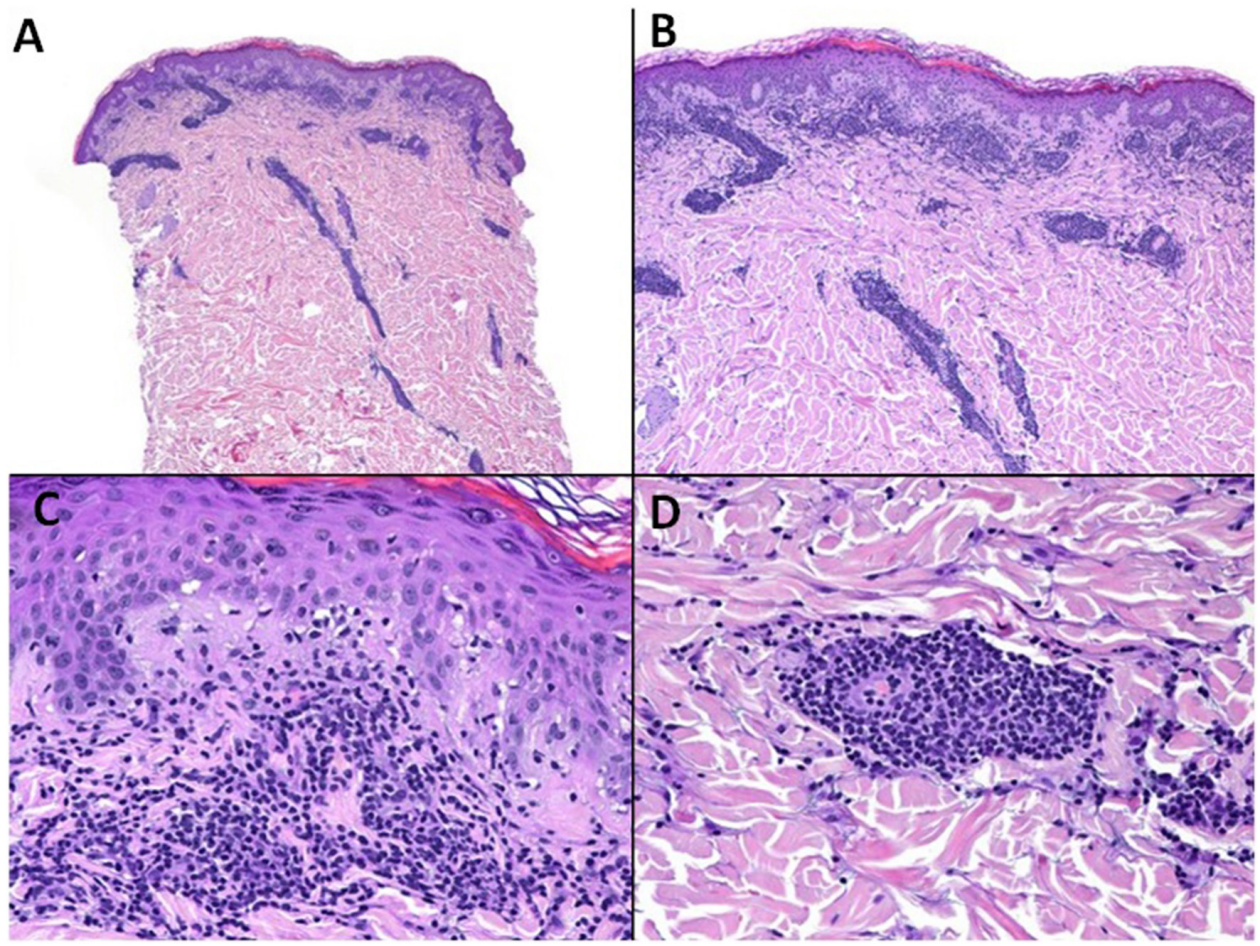
Spring eruption on the elbows. Histopathologic features (Case 1). Haematoxylin-eosin **(A)** (x10) **(B)** (x40) **(C)** (x200) **(D)** (x200). **(A, B)** Spared epidermis. Superficial and deep perivascular lymphocytic infiltrate. **(C)** Respective dermo-epidermal junction. **(D)** Infiltrate clustered around vessels.

In all cases, histological examinations revealed perivascular superficial and deep lymphocytic infiltrates in the dermis, along with variable degrees of dermal edema. Epidermal changes were present in four of the patients, along with mild spongiosis and parakeratosis (Figure 4). The anti-CD123 marker staining was negative for all patients (images not shown).

## Discussion

This study demonstrated the involvement of UVA radiation in skin lesions in a series of patients with summer and spring eruptions on the elbows, confirming the diagnosis by histopathological analysis.

PLE is a photo-induced eruption that is mainly observed in temperate latitudes. It is more severe in spring and summer, affecting mostly middle-aged women. This coincides with our series of patients, in which four of the five patients were women with a mean age of 39 years.

PLE affects the sun-exposed areas of the neck, the pre-sternal region, arms, forearms, and hands, and it often spares the face due to the hardening phenomenon. The lesions are polymorphous and may present as papules, vesicles, plaques, and nodules, which appear hours or days after exposure to the sun and remain for several days.

In our study, all five patients developed characteristic PLE lesions only on the elbows, while none of the patients developed photoprovocation of lesions on the back. At the initial visit prior to conducting photobiological studies, all patients described a cluster of papular lesions that appeared several hours/days after sun exposure and were located exclusively on the elbows, bilaterally in all cases. The clinical and histological data of this study are consistent with those of the authors who described spring and summer eruptions on the elbows as a localized variant of PLE (4). However, contrary to our research, these authors retrospectively described the lesions appearing in the patients, and they were unable to confirm the entity by photobiological studies. Our study demonstrated, for the first time, the generation of summer PLE lesions on the elbows by irradiation of the skin with repeated and increasing dose series of UVA radiation. The photobiological study is beneficial to the study of photodermatoses as it allows for the confirmation of light involvement in certain pathologies. In our study, all patients had a normal MED with no abnormal reaction to UVA 24 h after the phototests, which is in agreement with other authors (16). In the case of PLE, the photoprovocation test involving repeated phototests on the skin provides a great deal of information, as it allows skin lesions to be reproduced in a controlled manner using different light sources; this not only confirms the diagnosis but also provides information on the spectral band that generates the pathology in each patient. In most of the published series of photobiological studies on PLE with photoprovocation of lesions, UVA (320–400 nm) has been shown to be more effective than UVB, coinciding with the results observed in our patients whose lesions were photoprovoked with UVA (17–19). A limitation of our study was that no full photoprovocation tests were performed. We did not expose the patients to UVB or total UV solar-simulated irradiation to investigate the possible implication of UVB in the generation of this variant of PLE. The reason was that, after our experience with many normal photoprovocation tests for patients with normal PLE, repeated doses of UVB over several days only promoted high erythema in the patients' skin but did not reproduce lesions that were provoked only under UVA.

An advantage of the photoprovocation test is that, in the reproduction of lesions under controlled UVA exposure, the lesions can be biopsied, and the results can be confirmed by histopathological studies. In this regard, the photobiological study in our series of patients was particularly useful as it allowed us to confirm the involvement of UVA light in the appearance of lesions on the elbows and, therefore, to consider the condition as a variant of PLE, as previously described (4).

PLE diagnosis is usually clinical and is based on typical clinical history and characteristic skin manifestations. There are no specific diagnostic laboratory tests for PLE, and they are usually performed to rule out other dermatoses, e.g., photosensitive lupus erythematosus. This differential diagnosis should be made with summer and spring rashes on the elbows, as similar lesions on the elbows have been described in patients with lupus erythematosus (20). Contrary to summer and spring eruptions on the elbows as a PLE variant, patients with lupus erythematosus usually present lesions in other parts of the body, often accompanied by residual lesions and other histological features such as epidermal atrophy, vacuolar degeneration, or mucin deposits, among others. Contrarily, the authors who described summer and spring eruptions on the elbows highlighted a significant feature, i.e., the negative immunohistochemistry for CD123 in all patients in their series (4). These findings are consistent with our study's results, which also showed negative anti-CD123 staining in all patients, even though it has been recently published that CD123 is a useful marker for differentiating cutaneous lupus erythematosus and polymorphous light eruption from pityriasis rosea and mycosis fungoides (21).

To help clarify the involvement of UVA radiation and to ensure the definitive diagnosis of this entity, photoprovocation testing is a key element. To our knowledge, this was the first time that photobiological testing was performed in this PLE variant, which exclusively affects the elbows. It is likely that this entity has been underdiagnosed due to its peculiar clinical presentation and the difficulty of performing photobiological studies in many healthcare centers.

## Data availability statement

The raw data supporting the conclusions of this article will be made available by the authors, without undue reservation.

## Ethics statement

The studies involving humans were approved by Malaga Province Ethics Committee (EC:16-12-21-Consejería de Salud, Servicio Andaluz de Salud, Junta de Andalucía, Spain). The studies were conducted in accordance with the local legislation and institutional requirements. The participants provided their written informed consent to participate in this study. Written informed consent was obtained from the individual(s) for the publication of any potentially identifiable images or data included in this article.

## Author contributions

MG: Conceptualization, Investigation, Project administration, Resources, Writing—original draft. RC-M: Conceptualization, Formal analysis, Investigation, Methodology, Writing—review and editing. NL-N: Data curation, Investigation, Validation, Writing—review and editing. PL-J: Investigation, Methodology, Validation, Writing—review and editing. EN-dG: Data curation, Investigation, Methodology, Software, Writing—review and editing. RB-G: Investigation, Methodology, Validation, Writing—review and editing. EH-A: Investigation, Methodology, Validation, Visualization, Writing—review and editing. JA: Conceptualization, Investigation, Methodology, Writing—review and editing.
